# Sustainable Ecosystem Management Strategies for Tackling the Invasion of Blackchin Tilapia (*Sarotherodon melanotheron*) in Thailand: Guidelines and Considerations

**DOI:** 10.3390/ani14223292

**Published:** 2024-11-15

**Authors:** Thotsapol Chaianunporn, Thitipong Panthum, Worapong Singchat, Kanokporn Chaianunporn, Warong Suksavate, Aingorn Chaiyes, Narongrit Muangmai, Dokrak Marod, Prateep Duengkae, Kornsorn Srikulnath

**Affiliations:** 1 Department of Environmental Science, Faculty of Science, Khon Kaen University, 123 Mittraphap Road, Muang District, Khon Kaen 40002, Thailand; thotsapol@kku.ac.th; 2 Animal Genomics and Bioresource Research Unit (AGB Research Unit), Faculty of Science, Kasetsart University, Bangkok 10900, Thailand; thitipong.pa@ku.th (T.P.); worapong.singc@ku.ac.th (W.S.); chaiyes.stou@gmail.com (A.C.); ffisnrm@ku.ac.th (N.M.); fforptd@ku.ac.th (P.D.); 3 Special Research Unit for Wildlife Genomics (SRUWG), Department of Forest Biology, Faculty of Forestry, Kasetsart University, 50 Ngamwongwan, Chatuchak, Bangkok 10900, Thailand; fforwos@ku.ac.th; 4 Faculty of Medicine, Mahasarakham University, Mueang Maha Sarakham District, Maha Sarakham 44000, Thailand; kanokporn.s@msu.ac.th; 5 Department of Fishery Biology, Faculty of Fisheries, Kasetsart University, Bangkok 10900, Thailand; 6 Department of Forest Biology, Faculty of Forestry, Kasetsart University, 50 Ngamwongwan, Chatuchak, Bangkok 10900, Thailand; dokrak.m@ku.ac.th; 7 Department of Genetics, Faculty of Science, Kasetsart University, Chatuchak, Bangkok 10900, Thailand; 8 Center for Advanced Studies in Tropical Natural Resources, National Research University-Kasetsart University, Kasetsart University, Bangkok 10900, Thailand; 9 Biodiversity Center Kasetsart University (BDCKU), Kasetsart University, Bangkok 10900, Thailand

**Keywords:** invasive species, blackchin tilapia, *Sarotherodon melanotheron*, management strategies, ecological impact, biological control, genetic biocontrol

## Abstract

The invasion of blackchin tilapia in Thailand is a critical ecological threat affecting native biodiversity and destabilizing ecosystems. It is also resulting in significant ecological and socio-economic impacts. Originally from West Africa and introduced globally for aquaculture, blackchin tilapia thrive in Thailand’s brackish and freshwater ecosystems because of their high reproductive rates, environmental adaptability, and trophic plasticity. This review evaluates potential solutions for the invasion of blackchin tilapia in Thailand from a scientific perspective. The successful invasion of blackchin tilapia in Thailand can be attributed to several key factors, including late detection of the species, the delayed government response, the absence of effective management strategies, and the species’ high invasive potential. Given its wide distribution, large population size, and high reproductive rate, the eradication of the entire blackchin tilapia population is unlikely. Management efforts should focus on containment and impact reduction. Early detection and monitoring at the invasion front remain crucial to limit the species’ spread. Developing an online platform for tracking invasive species would aid these efforts. Public education and outreach are essential for promoting responsible behaviors for preventing further spread. Encouraging the utilization of blackchin tilapia as a food source and for commercial purposes could help suppress its population while supporting local economies. Biological control and other potentially harmful strategies should be avoided unless supported by strong scientific evidence to ensure minimal risks to ecosystems. Scientific research into controlling blackchin tilapia should be encouraged to develop suitable strategies, including using sterile triploid tilapia. The “polluter pays” policy should be implemented to cover the costs associated with invasive species. Coordinated efforts between researchers, policymakers, and the public are crucial for formulating effective management strategies to promote a balance between economic benefits and long-term environmental sustainability.

## Simple Summary

Blackchin tilapia, a fish native to West Africa, is causing significant ecological and economic problems in Thailand after being introduced for aquaculture. It reproduces rapidly and thrives in local brackish and freshwater habitats, posing a threat to native species and disrupting ecosystems. This review examines possible strategies for managing the blackchin tilapia invasion in Thailand. Given its adaptability and high reproductive rate, complete eradication is unlikely, so management efforts should focus on controlling its spread and minimizing its impact. Early detection and continuous monitoring are key to mitigating its effects, and the development of an online platform for tracking invasive species can be beneficial. Public education on preventing further spread is essential. Promoting the use of blackchin tilapia as a food source or other commercial product may help control its population while benefiting local economies. Risky strategies like biological control and genetic biocontrol should only be pursued with strong scientific evidence. Effective and sustainable management will require collaboration between researchers, policymakers, and the public.

## 1. Introduction

The term tilapia describes a group of tropical freshwater fish in the family Cichlidae (including *Oreochromis*, *Tilapia*, and *Sarotherodon* species) native to Africa and the southwestern Middle East [1,2,3,4]. Since the 1930s, they have been introduced globally for purposes such as aquaculture, ornamental trade, baitfish, and biological control, with increasing market demand in developed countries. Some tilapia, particularly Nile tilapia (*Oreochromis niloticus*) and spotted tilapia (*Pelmatolapia mariae*), are invasive species with significant impacts on non-native aquatic ecosystems worldwide, such as reducing the populations of native fish species, particularly through competition and hybridization and biodiversity losses in freshwater ecosystems [5,6]. Their ability to adapt to diverse environments, rapid reproduction rates, and broad diets contribute to their success as invasive species [5].

Blackchin tilapia (*Sarotherodon melanotheron*) has been reported as an invasive species in various regions, including Manila Bay, the Philippines [7], and the Indian River system, Florida [8]. This species, native to West Africa and found from Senegal to Zaire, is abundant in coastal lagoons, earning the name “West African lagoon tilapia” [9]. Three subspecies were identified using allozyme analysis: (1) *S. melanotheron heudelotii* from Senegal to Sierra Leone, (2) *S. melanotheron melanotheron* from Côte d’Ivoire to southern Cameroon, and (3) *S. melanotheron nigripinnis* from Equatorial Guinea to the Congo River mouth [10]. They are notable for their exceptional adaptability to a wide range of salinities and oxygen availability and tolerate overcrowding under certain conditions [11,12,13,14]. This species exhibits paternal mouthbrooding behavior in which the male churns eggs in his mouth for 14–18 days after fertilization [15,16]. This behavior can reduce egg mortality and increase the reproductive rate of blackchin tilapia. Both juvenile and adult blackchin tilapia are primarily planktivorous, consuming a wide range of plankton and other prey items such as the Cyanophyceae, Chlorophyceae, Bacillariophyceae and Rotifera, and Cladocera [17,18]. Their wide environmental tolerance, trophic adaptability, and high reproductive rates make them a successful invasive species. Although primarily brackish, they can live in freshwater and seawater bodies [19,20].

**Figure 1:**
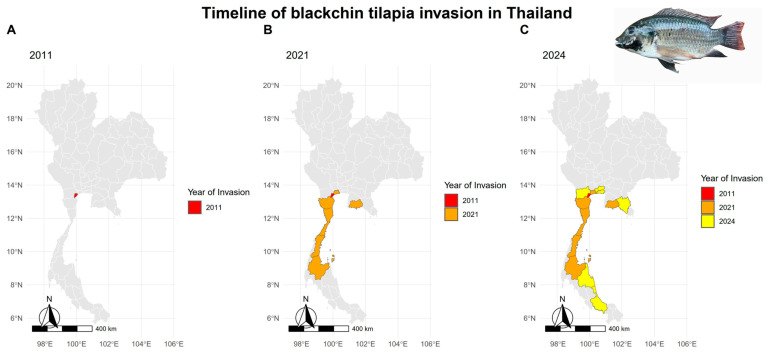
Timeline of blackchin tilapia (*Sarotherodon melanotheron*) invasion in Thailand. This figure illustrates the progressive invasion of blackchin tilapia across different provinces of Thailand from 2011 to 2024. Each map highlights the provinces invaded by blackchin tilapia by the indicated year, with consistent color coding to represent the invasion year (**A**) 2011—red; (**B**) 2021—orange; (**C**) 2024—yellow [21,23].

Blackchin tilapia (*S. melanotheron*) was introduced to Thailand’s freshwater ecosystems in 2010s. However, the origin of the blackchin tilapia invasion in Thailand remains unclear due to conflicting reports. Two thousand blackchin tilapia were legally introduced by a private company in 2010 for breeding research [21]. Although the company claimed that all imported fish were destroyed, it is possible that some fish might have escaped from the cage since DNA testing by the Department of Fisheries linked the invasive populations to one single parent stock [22], raising concerns about compliance with biosecurity protocols. The spread of blackchin tilapia in Thailand was first recorded in 2011 in Samut Songkhram Province. From 2020 to 2021, the species continued to spread, with widespread outbreaks expected in central, eastern, and southern regions, including Samut Sakhon, Rayong, Phetchaburi, Prachuap Khiri Khan, Chumphon, and Surat Thani Province [23]. Now, the invasive species has since spread to 13 provinces, including Chanthaburi, Rayong, Samut Prakan, Samut Songkhram, Samut Sakhon, Bangkok, Ratchaburi, Phetchaburi, Prachuap Khiri Khan, Chumphon, Surat Thani, Nakhon Si Thammarat, and Songkhla [24] (Figure 1).

The introduction of blackchin tilapia in Thailand is resulting in significant ecological and socio-economic impacts [25,26]. Their dominance in brackish habitats has caused declines in indigenous fish populations, disrupted ecological balance, and reduced biodiversity by overgrazing aquatic vegetation and outcompeting native species for food and habitat. Invasive blackchin tilapia have long been a significant concern for commercial aquaculture, particularly in fish and shrimp farms, where they cause substantial damage by consuming fry, roe, and other important food sources like phytoplankton and zooplankton, which are critical for the development of fish and shrimp. Thai shrimp farmers and coastal fishermen have complained regarding the detrimental impact of this species for several years [26]. In addition, similar to other invasive tilapias, the presence of blackchin tilapia potentially deteriorates water quality, including promoting sediment resuspension, increased turbidity, altered nutrient levels, and enhancing eutrophication [27]. These effects are due to the species’ trophic plasticity, adaptability, hardiness, and multiple spawning events. This species disrupts food web dynamics and nutrient cycling, destabilizing ecosystems. Moreover, the introduction of blackchin tilapia into aquatic ecosystems might have significant genetic effects on native fish populations. These effects can be categorized into direct impacts, such as hybridization, and indirect impacts, such as a decline in population size and loss of genetic diversity in native species. Despite possible socio-economic benefits, blackchin tilapia is highly invasive and potentially problematic beyond its native range [28].

In response to this ecological crisis, a series of measures have been implemented by the Thai government and the Department of Fisheries to control the spread of blackchin tilapia. The Ministry of Agriculture and Cooperatives has prioritized this issue as a national concern [29]. A committee has been established to address the outbreak and implement five key measures: (1) controlling and eradicating blackchin tilapia in all affected water sources; (2) releasing predatory fish, such as Asian sea bass (*Lates calcarifer*) and other tilapia species, to manage the invasive population; (3) utilizing captured blackchin tilapia for commercial purposes, including fish powder production; (4) continuously monitoring and surveying natural water sources to track the spread of the species; and (5) conducting public awareness campaigns to encourage community involvement in eradication efforts.

However, many of these measures lack a strong scientific foundation and may not provide long-term solutions. While public awareness campaigns are valuable, they must be supported by rigorous scientific research and continuous monitoring. Decisions about blackchin tilapia invasions often involve speculation, with advocates and opponents sometimes overstating their positions. Although various control measures have been implemented, their efficacy remains uncertain. The invasion of blackchin tilapia in Thailand highlights the urgent need for scientifically sound management strategies based on comprehensive ecological assessments. Therefore, this review evaluates potential solutions to address the invasion of blackchin tilapia, assessing both measures proposed by the Thai government and other possible options from a scientific perspective.

## 2. Challenges to the Management of Invasive Blackchin Tilapia in Thailand

### 2.1. Is It Still Possible to Eradicate the Entire Population of Invasive Blackchin Tilapia from Thailand?

Bernery et al. (2022) [30] proposed a two-stage management strategy based on the invasion process: (1) prevention, early detection, and monitoring, and (2) eradication, containment, and suppression. If invasive blackchin tilapia is detected early and the invasion area is very limited, complete eradication of the population may still be financially and logistically feasible. However, it might not be possible in this situation because the current distribution of blackchin tilapia spans over 300 km^2^ across 13 provinces in Thailand (Figure 1), with populations numbering in the millions [31,32]. Despite this, monitoring and early detection remain critical measures for limiting the impact of blackchin tilapia, including its potential spread to neighboring countries such as Malaysia and Cambodia.

In the past, a significant barrier to managing blackchin tilapia was the general lack of public awareness and understanding of the ecological impact of invasive species. Many communities might not recognize the threats posed by blackchin tilapia, especially when the species has economic or cultural value, such as in local fisheries or as a food source. This lack of awareness can result in resistance to control measures and noncompliance with regulations designed to prevent the spread of the species [33,34]. For example, in communities with low awareness of invasive fish, resistance to control efforts may arise [35]. In Thailand, public awareness of the invasion of blackchin tilapia has historically been low, likely due to its similarity to Nile tilapia (*Oreochromis niloticus*), a key economic species in the country [36]. However, by 2024, public attention has significantly increased as a result of the socio-economic impacts of blackchin tilapia, amplified by media and social media efforts. Consequently, people are becoming more adept at identifying blackchin tilapia and recognizing the threat it poses. To prevent further invasion and enhance monitoring of this species, public education and outreach must be prioritized to build support for control efforts and promote responsible behaviors, such as refraining from releasing blackchin tilapia into new habitats and reporting sightings of the species in previously uncolonized areas. Engaging the public in citizen science initiatives against invasive species can significantly contribute to the success of long-term management strategies. An online platform for reporting blackchin tilapia and other invasive species in Thailand should be created to serve as a tool for tracking and managing invasive species, ultimately contributing to more effective conservation efforts.

To enhance management efforts, ecological models, including species distribution and population dynamics models should be developed because they are essential for understanding the drivers behind blackchin tilapia expansion, identifying areas at risk of invasion and planning effective long-term range-wide control strategies. These models require accurate environmental data, species traits, and biotic interactions, sourced from comprehensive databases and field studies. By using model predictions to explore best- and worst-case scenarios, these insights can inform targeted management actions, such as physical barriers, selective removal, and habitat restoration. Proactive, adaptive management plans can then be implemented to mitigate the impact of blackchin tilapia on Thailand’s aquatic ecosystems.

### 2.2. Biological Control of Blackchin Tilapia

Managing blackchin tilapia is challenging because of its prolific reproductive capacity, large number of offspring, and high juvenile survival [9,37]. Rapid reproduction allows populations to replenish quickly after control efforts. Breeding occurs multiple times annually, possibly every few weeks, under favorable conditions. High fecundity enables rapid population recovery, even for a few survivors, necessitating sustained and intensive management. In addition, blackchin tilapia is characterized by a broad environmental tolerance and thrives in freshwater, brackish, and marine systems [19,20]. Due to its wide distribution range and large population size, chemical control methods, such as the use of rotenone or cyanide, are impractical as they could pose significant environmental risks (discussed below). Biological control, which involves using natural predators, parasites, pathogens, or competitors to manage invasive species, is one interesting option because it offers several advantages over other control methods like chemical, mechanical, or physical controls; for example, biological control minimizes chemical use, and once established, biological control agents can reproduce and maintain their populations, providing long-term, cost-effective, sustainable control without the need for frequent reapplications [38].

However, among the five key measures of the Thai government, the most concerning is the release of predatory fish, such as Asian sea bass (Barramundi—*Lates calcarifer*), to manage the invasive population because the selection of this biological agent was mainly based on its habitat preference (coastal waters, estuaries, and lagoons) being similar to that of blackchin tilapia [39] and its generalist feeding behavior [40]. They are expected to feed on fry and the roe of blackchin tilapia. In August 2024, the first lot of 30,000 adult Asian sea bass were released into the Tha Chin River in Samut Sakhon Province to control blackchin tilapia [41]. The question remains whether the Asian sea bass will prove to be an effective biological control agent.

The control of blackchin tilapia by Asian sea bass may not provide a long-term control because Asian sea bass is a generalist predator, and the two species did not coevolve. Without a shared evolutionary history, the predator–prey dynamics between sea bass and blackchin tilapia may not be well matched. Sea bass may not effectively prey on blackchin tilapia because of differences in behavior, microhabitat preferences, and other ecological interactions lacking in non-coevolved predator–prey pairs. In addition, the introduction of Asian sea bass may affect non-target species, leading to potential ecological imbalances. Moreover, this approach risks altering the natural biological community, including species, ecological, and genetic diversity. If not carefully managed, releasing predatory fish can disrupt local ecosystems. This can ultimately lead to ‘invasional meltdown’ (the introduction of one invasive species facilitates the establishment and spread of additional invasive species [42]) of native ecosystems. Thus, we suggested that it is important to rapidly evaluate the efficacy of Asian sea bass as a biological control agent against blackchin tilapia and its impact on non-target species and ecosystems before releasing additional batches.

In contrast, when another specialist predator of blackchin tilapia is introduced, the oscillating dynamics of predator–prey relationships pose significant risks to maintaining stable population levels, potentially leading to ecosystem destabilization and the extinction of the predator species. Moreover, this approach might be related to the potential introduction of another predator species whose ecological consequences are difficult to predict. Conversely, employing a generalist predator might affect non-target species in addition to blackchin tilapia. By contrast, we propose that introducing competitors, referred to here as “competitor-x”, may serve as one option of the effective biological control agents against blackchin tilapia, especially in closed water bodies, e.g., ponds, reservoirs or inland lakes. This species should not be a newly introduced species but rather one that is already present or native. Introducing species that compete with blackchin tilapia for resources can naturally limit tilapia population growth, thereby reducing its ecological impact. If competitor-x outcompetes blackchin tilapia, it might lead to a decrease in the population size or local extinction of the invasive fish. Competitor-x should inhabit the same environment as blackchin tilapia and exhibit high niche overlap, relying on similar resources, such as diet or habitat. Several fish species are candidates as competitors with blackchin tilapia. Species already introduced in Thailand such as Nile tilapia (*Oreochromis niloticus*) or native species such as walking catfish (*Clarias batrachus*) may be considered potential competitors of blackchin tilapia because they are found in similar habitats, are strong competitors, have high reproductive rates, and exhibit overlapping ecological niches (Nile tilapia [43] and walking catfish [44]).

Further research and trials are required to identify suitable natural enemies (predators, pathogens, or competitors) and assess their effectiveness against blackchin tilapia in aquatic environments in Thailand. A careful and rapid evaluation of the potential use of a biological control agent against blackchin tilapia should involve both experimental studies and field assessments to minimize ecological risks. First, controlled laboratory trials should be conducted to assess the predation or competitive efficiency of the biological control agent on blackchin tilapia across various life stages, focusing on their ability to control tilapia populations effectively without unintended impacts on non-target species. Concurrently, field studies in contained environments, such as isolated ponds or controlled sections of affected waterways, can be used to observe the interactions between the biological control agent and blackchin tilapia in more complex ecosystems. Additionally, ecological modeling could provide valuable insights into the potential outcomes of introducing the biological control agent on a larger scale, helping to predict longer-term effects and the likelihood of successful control. If preliminary results indicate high efficiency and low risk, further field applications could be considered, but these should be accompanied by ongoing monitoring and an adaptive management approach to respond to unforeseen ecological impacts promptly.

However, as the possession of live blackchin tilapia is banned everywhere in Thailand by the Thai government, such experiments are now impossible [24]. Therefore, the use of blackchin tilapia for scientific research, including the exploration of effective biological control strategies, should be supported under well-regulated measures.

### 2.3. Genetic Biocontrol Method of Blackchin Tilapia

**Figure 2:**
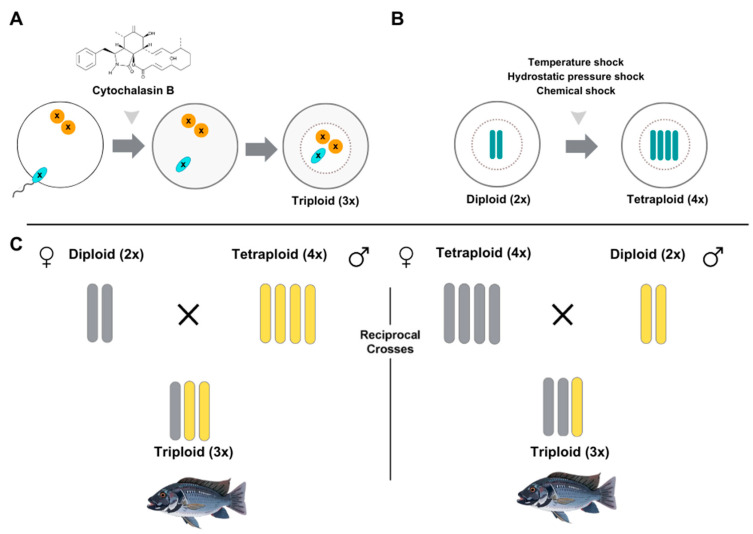
Methods for producing triploid fish. (**A**) Triploid fish are produced via physical treatments on diploid fish, while triploidy can be directly induced using physical or chemical (cytochalasin B) methods on fertilized eggs. (**B**) Tetraploid (4x) fish are generated from diploid (2x) fish using temperature shock, hydrostatic pressure shock, or chemical treatments (e.g., colchicine, cytochalasin B, 6-dimethylaminopurine, nitrous oxide). (**C**) Triploid fish are created by crossing tetraploid (4x) fish with diploid (2x) fish, resulting in sterile triploid (3x) offspring due to meiotic dysfunction.

Instead of employing other species as competitors, an alternative approach involves the introduction of sterile blackchin tilapia that satisfy many criteria for competitor-x. This method is called the ‘genetic biocontrol method’ [30,45] or ‘sterile-male approach’ [46]. The release of sterile males may reduce their reproductive resources, thereby competing for resources and potentially suppressing their reproductive success. This strategy can induce reproductive failure, aiding population control through resource competition. Methods such as polyploidization and genetic engineering have been proposed to develop sterile blackchin tilapia. Triploids, which are organisms with a third chromosome set added to the normal diploid set, can be induced by directly manipulating meiosis or by creating tetraploids (4x) and crossing them with diploids (2x) to yield triploid (3x) offspring (Figure 2). Tetraploid fish are created by manipulating early embryonic development in diploid fish through physical treatments, such as heat or pressure shock. Alternatively, triploidy can be induced in fertilized eggs through physical (heat, cold, or pressure shocks) or chemical (cytochalasin B) treatments to prevent the release of a second polar body, resulting in an extra set of chromosomes. These techniques are straightforward and cost-effective. Both methods are effective, with the 4x × 2x cross offering more control, and direct induction is more suitable for large-scale applications. For example, triploidy has been induced in catfish (*Pangasianodon hypophthalmus*) by heat shock [47], whereas caffeine treatment has been used to induce triploidy in African catfish (*Clarias gariepinus*). Triploid offspring are typically rendered sterile because of meiotic dysfunction, as an odd number of chromosome sets disrupt normal gamete formation. This is because unpaired chromosomal sets are retained, rather than separated, during cell division [48]. Sterility allows gonadal development but results in germ cells with abnormal chromosome numbers [49]. In fish, triploid males produce functional sperms with nonviable offspring, whereas triploid females have underdeveloped ovaries and retain their juvenile characteristics [50]. Triploid production has advanced to commercial viability in fish and bivalves, with significant progress in crustaceans. Triploidy enhances fishery production by reducing the energy required for gonadal growth and lowering the risk of escaped aquaculture stocks establishing feral populations. For example, in Atlantic salmon (*Salmo salar*), studies have shown that triploid fish exhibit different growth performances than diploids after being transferred to seawater [51]. Additionally, the risk of interbreeding between escaped farmed salmon and wild populations, which could threaten the genetic diversity of wild stocks, can be mitigated by triploidy [52,53].

Triploidy, a chromosomal manipulation technique that does not involve direct genetic modifications, is widely accepted in recreational fisheries and aquaculture with minimal public concern for pest control [45]. Research has indicated that triploid fish such as Atlantic salmon (*S. salar*) can achieve growth rates comparable to or even superior to those of diploids under certain conditions, making them a viable option for aquaculture [54]. Additionally, the reproductive capabilities of triploid fish, such as rainbow trout (*Oncorhynchus mykiss*), are reduced, minimizing the risk of breeding with wild populations if they escape [55]. Similarly, triploid oysters are used in aquaculture to enhance meat quality and production efficiency [56], demonstrating the versatility and effectiveness of this technique for different species. The induction of three chromosome sets renders fish sterile and prevents their reproduction.

This method is valuable for managing invasive species, such as blackchin tilapia, in Thailand by controlling population growth and mitigating ecological impacts. In established invasive species, releasing triploid males to mate with wild females can reduce population fertility. However, it includes potential challenges and ethical or ecological considerations. Although not tested in fish, insect studies suggest that large numbers of triploids may be needed to outnumber wild-bred males. For instance, studies on triploid mosquitoes have indicated that a substantial release is necessary to affect wild populations and reduce fertility [57]. Therefore, this approach may not be cost-effective for the large-scale control of blackchin tilapia and could lead to temporary high pest densities with significant ecological impacts. However, if implemented when pest populations are small, the required number of males might be manageable, and temporary ecological effects could be preferable to the long-term damage from widespread pests.

Although the technology used to produce all-female triploid fish populations is simple and applicable on a commercial scale, it does not always produce populations that are 100% triploid [54]. Fertility may sometimes occur in sterile females because of endoreplication at different ploidy levels, involving either the same or different genome types [58]. Gene editing or gene-drive techniques aimed at knocking out functional genes involved in sex development or fertility present promising alternative approaches, such as in insects [59]. However, the plasticity and complexity of the fish genome, along with the multiple functional gene systems that control sex determination and development, add challenges. Therefore, it is necessary to test this system and acquire basic structural and functional genomic information on blackchin tilapia.

### 2.4. Utilizing Captured Blackchin Tilapia for Commercial Purposes: Addressing True Sustainable Economic and Logistical Challenges

Due to the wide distribution range and large population size of invasive blackchin tilapia, large-scale control measures face economic and logistical challenges that complicate management. Mechanical removal methods for blackchin tilapia, such as netting and electrofishing, require significant financial and human resources and are labor-intensive [60,61]. Even though electrofishing has shown promise as a control measure in northern Australia, reducing mature Mozambique tilapia (*Oreochromis mossambicus*) populations by 87% over 33 months [62], a review of invasive fish management noted that the effectiveness and sustainability of large-scale mechanical fish removal are often limited by high costs and logistical difficulties [45]. These methods can be effective locally but are impractical for large water bodies or regions with extensive infestations. In addition, mechanical removal may disrupt non-target species and habitats, causing further ecological harm [63]. Chemical treatments, such as piscicides or cyanide (CN), can effectively reduce fish populations but are non-selective and may harm non-target species, including native fish and invertebrates. Research has shown that piscicides, while effective in controlling invasive fish, can have significant ecological impacts. For example, the use of rotenone, a common piscicide, reduces target fish populations but also harms non-target species, such as amphibians and invertebrates [64]. Similarly, using CN for fish control can damage coral reef ecosystems and affect non-target fish and invertebrates [65,66]. Moreover, the potential impact on water quality and environmental safety raises concerns, particularly in areas where treated water bodies are connected to larger ecosystems or used for drinking water or irrigation. Owing to potential ecological and health risks, careful consideration and regulatory approval are necessary for chemical treatments, which can be time-consuming and costly.

In July 2024, the Thai government announced a buying price of 15 baht (approximately 0.45 USD) per kg of blackchin tilapia during the peak of the outbreak [67]. Fishing authorities caught more than one million kilograms of blackchin tilapia between February and August 2024, and it was estimated that the outbreak of tilapia will cost the Thai economy at least 10 billion baht (293 million USD) [31]. The utilization of captured blackchin tilapia as food or for other commercial purposes, e.g., fish powder, fermented fish products, or fertilizer production, is a part of the government’s proposed measures. Given that the eradication of the entire blackchin tilapia population from Thailand is deemed impossible under the current circumstance as we discussed above, the exploitation of invasive blackchin tilapia as a food source or for other products could offer an effective sustainable way to suppress the invasive population and limit its spread [68]. Thai people have considerable experience dealing with various introduced and invasive species and have demonstrated an ability to adapt by utilizing these species as a food source, e.g., golden apple snail (*Pomacea canaliculata*) is now a part of Thai papaya salad [69], Nile tilapia (*O. niloticus*) has become an economically important species and protein source for Thai people [36], and common pleco fish (*Hypostomus plecostomus*) can be cooked as Pad Kra Pao (holy basil fish stir-fry), Kaeng som (Thai sour curry), grilled fish, or crispy fish wrap (pers. Obs.). However, this does not imply that the spread of blackchin tilapia should not be prevented, because this species continues to have devastating effects on native ecosystems. In addition, in the long term, offering high-price incentives for purchasing fish may also risk promoting the spread of blackchin tilapia if people begin rearing the fish in broader areas. Therefore, control measures should be implemented carefully to help minimize ecological risks.

Managing blackchin tilapia in Thailand requires a multifaceted approach to address its biological, economic, logistical, and social aspects. Strategies must be scientifically informed, well-funded, and supported by public engagement and policy frameworks. Continuous research and monitoring are essential for refining control methods, assessing their effectiveness, and minimizing ecological consequences. Learning from local and international experiences allows stakeholders to develop more robust and sustainable strategies for managing this invasive species and protecting Thailand’s aquatic ecosystems. The coordination of invasive species management requires cooperation among multiple stakeholders, including government agencies, non-governmental organizations, local communities, and international partners. Inconsistent policies, limited funding, and bureaucratic delays impede timely and effective responses to emerging invasions. Furthermore, the transboundary nature of aquatic ecosystems necessitates cross-jurisdictional cooperation in managing blackchin tilapia, which is difficult to achieve.

## 3. Perspectives

Tracing back to its origins, the introduction of blackchin tilapia into Thailand was primarily for aquaculture purposes. In the long term, several factors are expected to reduce the impacts of aquaculture on ecosystems and environment. These practices can lead to habitat destruction, genetic pollution, and water quality issues. Poorly managed aquaculture irreversibly alters aquatic ecosystems, diminishing the natural resource base crucial for aquaculture productivity. The escape of cultivated organisms is almost inevitable [30], making any cultured non-native organism a potentially invasive species, such as blackchin tilapia. The likelihood of the establishment of such species is extremely high, especially in tropical waters [70]. The open-water introduction of blackchin tilapia cannot proceed without further damage to native fish species and biodiversity. The impacts of species invasion are often confounded by habitat disturbance from human activities [71], such as dam construction, urban development, and deforestation, complicating the identification of causal factors in community changes in native fish or vegetation. Careful culture management practices are recommended in facilities that continue to raise blackchin tilapia or other potentially invasive species. Owing to the adaptability of blackchin tilapia [72], they should be raised in ponds without access to natural waters, preferably in regions with temperatures that prevent over-wintering in case of escape. Waste and effluent must be managed to avoid reaching natural water bodies. Investigating aquaculture facilities with no local establishment records of invasive species can help document the ‘best management practices’ or guidelines specific to blackchin tilapia aquaculture. In areas where non-native blackchin tilapia have not been introduced for culture or stocking, stringent efforts should be implemented to exclude them.

A “polluter pays” policy [73,74] is an essential regulatory approach and should be applied for managing and mitigating the impacts of bioinvasions caused by aquaculture practices. If evidence links a specific aquaculture company to the introduction and spread of invasive species like blackchin tilapia, it is both practical and equitable to hold the responsible party financially accountable for the resulting environmental and economic damage. By enforcing this policy, the company would be required to cover the costs associated with containment, mitigation, and restoration efforts to address the impacts of the invasive species. Additionally, implementing a “polluter pays” approach creates an incentive for companies to adopt preventive measures and adhere to best practices in biosecurity, ultimately reducing the risk of future invasions. Such a policy not only aligns with environmental accountability, but also promotes long-term sustainability by encouraging responsible management within the aquaculture industry.

In addition, research and investment in native species for aquaculture are urgently needed. Understanding the life history traits and growth performance of potential candidates is vital for managing proposed aquaculture programs. Mapping the distribution patterns of native species is necessary, and this information should be shared with government workers and farmers for aquaculture planning. For example, research on carp species, such as the common carp (*Cyprinus carpio*) and grass carp (*Ctenopharyngodon idella*), has demonstrated that aquaculture practices and sustainability can be significantly improved through detailed knowledge of their life history traits and distribution patterns [75,76]. There is a crucial need to develop local laws that mirror international guidelines and enforce them effectively to protect ecosystems from invasive species.

Local and national governments, along with international organizations, are obligated to invest in and promote aquaculture and stocking practices using non-disruptive fish species. Thus, policies should focus on utilizing native species and minimizing non-native introductions, particularly where endemic and threatened species exist. Although high-yield, low-cost protein sources offer only short-term benefits, environmental conservation is crucial for long-term sustainability. Careful planning and monitoring of aquacultural development is necessary. Promoting thoughtful management and oversight will balance the immediate benefits while preserving ecological integrity and biodiversity.

## 4. Conclusions

The invasion of blackchin tilapia in Thailand can be attributed to several causes, including late detection of the species, delayed government response, lack of management strategies, and the species’ high invasive potential. We assessed the Thai government’s five key measures and explored additional strategies, offering the following Do’s and Don’ts for sustainable ecosystem management for mitigating the spread and impact of blackchin tilapia in Thailand:

**Do’s**

- **Develop an online reporting platform:** create a system for tracking and managing invasive species, allowing the public and experts to report sightings of blackchin tilapia and other invasive species.
- **Promote public education and outreach**: engage the public in citizen science initiatives and promote responsible behaviors to prevent the spread of blackchin tilapia.
- **Ensure rapid response**: act swiftly upon the detection of invasive species to minimize their spread and ecological damage.
- **Develop ecological models**: utilize species distribution, environmental factors and population dynamic models to identify areas at risk of invasion and guide management efforts for blackchin tilapia.
- **Allow scientific research**: support the use of blackchin tilapia for scientific research, including exploring biological control and genetic biocontrol strategies.
- **Promote the use of blackchin tilapia for food and commercial purposes**: encourage the utilization of blackchin tilapia as a food source and for other commercial purposes, such as fish powder and fertilizer, to help control its population while benefiting local economies.
- **Enforce the polluter pays policy**: the government should require polluters to cover the costs associated with containment, mitigation, and restoration efforts needed to address the impacts of invasive species.

**Don’ts**

- **Expect to eradicate the entire population**: Avoid assuming that the entire population of blackchin tilapia can be eradicated. Given its wide distribution and high reproductive rate, eradication is unlikely, and management efforts should instead focus on containment and impact reduction.
- **Implement control without a scientific basis**: avoid control strategies, such as biological control, genetic biocontrol, or offering high-price incentives for fish purchases, unless they are strongly supported by scientific evidence accompanied by adequate control measures to ensure minimal ecological risk.
- **Use harmful control measures**: refrain from using control methods, such as chemical control, that could negatively impact the broader ecosystem.

The invasion of blackchin tilapia in Thailand poses a significant ecological threat, with far-reaching impacts on native biodiversity and ecosystem stability. The current status of this invasive species, its ecological impact, and management challenges are reviewed in this study. This review provides valuable insights into invasion scenarios and management but must be used cautiously due to inherent limitations and uncertainties, such as incomplete data due to the ban of blackchin tilapia possession in Thailand, unforeseen environmental changes, and complex species interactions that affect prediction accuracy. Despite these uncertainties, we propose that ecological models are crucial tools for policy and management decisions. The importance of early detection, rapid response, and continuous monitoring in managing invasive species has been underscored, along with the urgent need to implement robust management strategies. Understanding the relationship between economic choices and ecosystem health is critical, allowing economic incentives to prevent or limit the impacts of invasive species and safeguard both ecosystems and economies. Without intervention, the continued spread of blackchin tilapia could cause irreversible damage to the freshwater, estuarine, and marine ecosystems in Thailand and neighboring countries. Coordinated efforts involving researchers, policymakers, and the public are required to address this crisis. Further research and collaboration are essential to refine predictive models and develop comprehensive management strategies to protect Thailand’s aquatic ecosystems from invasive threats.

## Data Availability

No new data were created in this study.
